# Stakeholder consultations and the legitimacy of regulatory decision‐making: A survey experiment in Belgium

**DOI:** 10.1111/rego.12323

**Published:** 2020-06-11

**Authors:** Jan Beyers, Sarah Arras

**Affiliations:** ^1^ Department of Political Science University of Antwerp Antwerp Belgium

**Keywords:** agency, consultation, interest group, regulatory decision‐making, survey experiment

## Abstract

Agencies consult extensively with stakeholders such as industry associations, nongovernmental organizations, and trade unions. One rationale for consultations is that these improve procedural legitimacy and lead to greater acceptance of regulatory outcomes by citizens and the regulated industry. While this presumption of a positive relation between stakeholder consultations and the legitimacy of agencies is widespread, research analyzing this relationship remains scarce. Using a survey experiment, we examine the effect of open and closed consultations on the acceptance of procedures and regulatory outcomes in the field of environmental politics. The results demonstrate that consultation arrangements positively affect the acceptance of decision‐making procedures, especially when regulators grant access to different types of stakeholders. However, although the consultation arrangement itself does not directly affect acceptance of the regulatory outcome, procedural legitimacy matters, as it increases decision acceptance among individuals who are negatively disposed toward government regulation.

## 1 Introduction

In recent decades, electorally accountable institutions – parliaments and governments – have delegated important competencies to independent regulatory agencies (“agencies” hereafter) (Levi‐Faur 2005). For example, in the European Union (EU), more than 30 agencies have been established since 1975, and in the United States (US), agency decision‐making dwarfs ordinary legislation (West 2004; Furlong & Kerwin 2005; Wonka & Rittberger 2010; Egeberg & Trondal 2017). Much of this regulatory work is situated in sensitive areas, such as the regulation of pharmaceuticals, banking, and food safety. This delegation of policymaking responsibilities from traditional representative institutions to agencies may limit the input of citizens and companies which are affected by the regulatory decisions; this raises concerns about accountability and legitimacy. Hence, both American and European scholars have identified the reliance on agencies to potentially reinforce the emergence of a democratic deficit (West 2004; Busuioc 2009).

Considerable research has been conducted on how agencies seek to derive legitimacy from stakeholder consultations (Majone 1999; Damonte *et al*. 2014; Yackee 2014; Braun 2012). Consultations are procedures initiated by agencies to grant access to stakeholders such as nongovernmental organizations (NGOs), companies, business associations, and professional organizations. Legislators in the US and the EU have established various institutional devices requiring agencies to consult stakeholders and the general public (Arras & Beyers 2019; Beyers & Arras 2019; Furlong 1997; Borrás *et al*. 2007; Yackee 2014). For instance, in the US, the Administrative Procedure Act stipulates that agencies actively solicit stakeholder input, while the Federal Advisory Committee Act governs more than 800 advisory committees that make recommendations to federal agencies (West 2004; Moffitt 2010). Similarly, the publication of the White Paper on European Governance in 2001 by the European Commission (EC) marked the emergence of an extensive consultation regime that includes the widespread use of open consultations, as well as advisory committees (Quittkat 2011; Rasmussen & Carroll 2014; Rasmussen & Gross 2015; Bunea 2017).

Most research on how agencies interact with stakeholders is situated at the agency level and analyzes the control mechanisms governing the delegation of competencies (Busuioc 2009; Rittberger & Wonka 2011; Egeberg & Trondal 2017), to what extent consultation regimes benefit regulated industries (Furlong 1997; Furlong & Kerwin 2005), how consultation participants influence regulatory outcomes (Furlong 1997; Yackee 2006; Yackee & Yackee 2006; West 2009; West & Raso 2013), and how consultations facilitate legislative oversight (McCubbins & Schwartz 1984; Kelemen 2002; Font & Pérez Durán 2016; Arras & Braun 2018) or enhance agency reputations (Moffitt 2010). However, the perspective of individual citizens and companies which face regulatory costs has hardly been considered in this field (Borrás *et al*. 2007; but see Grimmelikhuijsen *et al*. 2019). Therefore, an important research question is whether agency consultations may improve legitimacy perceptions among recipients of regulatory policies. To answer this question, we build on a considerable body of recent experimental political science research, analyzing the relation between public policymaking and legitimacy perceptions (de Fine Licht 2014a; Esaiasson *et al*. 2016; Grimmelikhuijsen *et al*. 2019).

Specifically, by conducting a survey experiment, we analyze how two types of consultation procedures by agencies, namely advisory committees and online public consultations, affect procedure acceptance and the acceptance of regulatory outcomes by citizens and regulated companies. We thus test the extent to which citizens and the regulated industry believe that “decision‐makers have the right to make the decisions and that these decisions should be accepted” (de Fine Licht 2011, p. 185). The experiment focuses on a regulation abolishing the use of plastics in food packaging. Such regulation is potentially controversial among both citizens and companies. Although there might be diffuse benefits such as a less polluted environment, citizens and companies bear costs as the latter face an expensive transition to new production processes, which may result in higher consumer prices for the former. Consultations could be used to cope with these tensions and to establish policies that gain widespread support. Our experimental tests demonstrate that consultations generate a positive impact on the propensity to accept a decision‐making procedure, especially when regulators explicitly balance different interests by granting access to both affected industries and consumer interests. Although the consultation arrangement itself does not directly affect acceptance of the regulatory outcome, accepting the adopted decision‐making procedure matters, as this increases the decision acceptance among individuals who are negatively disposed toward government regulation.

## 2 Legitimacy, consultations, and agency decision‐making

Agencies have far‐reaching competences that directly affect citizens and companies, they operate in complex areas in which knowledge is contested, and many of their decisions create winners and losers (Borrás *et al*. 2007).All this raises questions of accountability and legitimacy (West 2004; Furlong & Kerwin 2005; Busuioc 2009; Rittberger & Wonka 2011). Hence, the idea that agencies are apolitical is not only naive but also fails to square with the facts. As West argues with respect to the US context, technical topics “are not neatly separable from the political issues that frequently dominate bureaucratic policymaking” (2004, p. 75). Regulatory outcomes are often controversial and may conflict with the preferences of important societal segments; therefore, a crucial prerequisite for legitimacy is that policymaking procedures and outcomes are experienced as fair and just.

The paper adopts a multi‐faceted conceptualization of legitimacy and distinguishes three dimensions (Scharpf 1999; Schmidt 2013; Strebel *et al*. 2018). The first two dimensions concern policymaking procedures and emphasize input‐ and throughput‐legitimacy. Highlighting *input‐oriented legitimacy* requires that citizens and regulated companies, or their representatives, can participate in policymaking processes (Scharpf 1999). Nonetheless, participation opportunities might be perceived as limited if these do not correspond with procedures that are experienced as efficient, accountable, transparent, and inclusive. *Throughput legitimacy* presumes that the diversity of the consulted stakeholders, the possibility for deliberation among these stakeholders and the overall transparency of the policymaking process lead to policies that are more widely accepted (Schmidt 2013). This understanding of procedural legitimacy bears a resemblance to the concept of procedural justice in the criminological and psychological literature which analyzes how the quality of the policymaking processes and the nature of policy implementation affects legitimacy beliefs and shapes compliance among individuals (Thibaut & Walker 1975; Tyler 1994, 2006; Murphy *et al*. 2009; Gifford & Reisig 2019).

In contrast to these procedural notions, one might also presume that policymaking is primarily driven by the need to establish effective policies; “outputs” are key ingredients of legitimacy and procedures are primarily seen as the means to an end (Majone 1999; Scharpf 1999). If the dimension of *output‐oriented legitimacy* is crucial, consultation arrangements matter less for how targets of regulations assess regulatory outcomes. Output‐oriented legitimacy entails that citizens and the regulated industry care primarily about how the effectiveness and problem‐solving nature of agency policy outputs align with their political views, much less about participation (*input*) or the nature (*throughput*) of policymaking procedures. The notion that output legitimacy matters more than procedural legitimacy resonates with scholarship in the procedural justice literature claiming that the extent to which policies are consistent with a person's values, for instance the extent to which costs and benefits are fairly distributed, is more relevant than procedural fairness judgments (Murphy *et al*. 2009; Gifford & Reisig 2019, p. 384).

The central presumption of a procedural perspective is that consultations can positively affect legitimacy, because they contribute to a positive assessment of the policymaking procedures and a greater acceptance of policies. Although this positive relation is often assumed and seldom empirically studied (exceptions include Agné *et al*. 2015; Bernauer & Gampfer 2013; Bernauer *et al*. 2016), policymakers explicitly refer to the assumption of increased legitimacy as a driver behind consultation arrangements (Arras & Braun 2018; Quittkat & Kohler‐Koch 2013). Transparency about stakeholder involvement, which is part of mechanisms fostering throughput legitimacy, informs regulatees about how policymakers take decisions, how policymakers deliberate and vote, the extent to which stakeholders are consulted, and how evidence obtained from these consultations informs their decisions.

Three distinct theoretical accounts underpin this positive relationship (Schmidt 2013). First, consultations allow stakeholders to monitor agencies, gather information, and disseminate this information to the broader public. This monitoring is what principal agency theorists call “fire alarm oversight”(McCubbins & Schwartz 1984; Kelemen 2002); the input from stakeholders stimulates agencies to adopt transparent procedures, making it easier not only for principals but also for the public and journalists, for instance, to hold agencies accountable for their conduct. Second, deliberative democratic theory presumes that the information stakeholders provide during consultations stimulates deliberation and improves the agencies' problem‐solving capacity. High‐quality throughput makes policymakers more acquainted with a more heterogeneous set of stakeholders and ensures that alternative viewpoints are heard (Agné *et al*. 2015; de Fine Licht et al. 2016). Third, procedural fairness theory, largely developed in social psychology and legal studies, presumes that fair procedures – such as hearing all affected parties – will produce outcomes that are more widely accepted (Thibaut & Walker 1975; Tyler 1994, 2006). In short, consultations ensure that citizens and companies perceive decision‐making procedures as fair and just, which makes them more likely to accept regulatory outcomes even if they do not favor these outcomes.

Recently, a substantial body of experimental research in political science has analyzed the relation between procedures and legitimacy perceptions. All these studies distinguish between the propensity to judge procedures and decision outcomes as acceptable, fair, and just (i.e. procedure acceptance and decision acceptance) as two important aspects of legitimacy perceptions and analyze their relation with the decision‐making procedure that was followed. The main hypothesis is that transparent procedures accounting for a broad array of preferences enjoy higher levels of procedure acceptance and positively affect decision acceptance.

A first set of experimental studies focusing on transparency found mixed results. For instance, evidence on how decisions were made (“transparency in the process”) – by elected politicians, policy experts, or via the participation of citizens – had no effect on procedure acceptance, decision acceptance, or general political trust (de Fine Licht 2011; Grimmelikhuijsen 2012; Grimmelikhuijsen & Meijer 2012; Grimmelikhuijsen *et al*. 2013). Other experiments have demonstrated more positive effects of transparency, especially transparency measures explaining the rationale of a policy (de Fine *et al*. 2014; de Fine Licht 2014b). However, transparency in process, for instance the disclosure of detailed information on decision‐making processes, did not increase perceived legitimacy substantially; and in some instances, it even exacerbated legitimacy perceptions.

Some field and survey experiments have focused explicitly on how decision‐making procedures shape procedure and decision acceptance (Esaiasson *et al*. 2012, 2016, 2017; de Fine Licht 2014a; Arnesen 2017; Porumbescu & Grimmelikhuijsen 2018; Strebel *et al*. 2018). Typical of these experiments is the manipulation of the objective decision‐making procedure and the actual decision; the acceptance of procedures and decisions is measured post‐treatment. For instance, subjects are informed that particular decisions are made by elected politicians in city councils, by experts in an agency, or through citizen referenda. One general conclusion runs through all these studies, namely, increasing decision acceptance is not simply a matter of designing more transparent or open decision‐making procedures, because individuals have intrinsic preferences about policy outputs irrespective of decision‐making procedures.

Finally, we are aware of two survey experiments in the field of global climate politics, one among Indian and US citizens (Bernauer & Gampfer 2013) and another among Chinese citizens (Bernauer *et al*. 2016), that test how consultations affect legitimacy perceptions among citizens. Both studies have confirmed that consulting business associations and civil society groups strengthened procedural acceptance. Interestingly, Bernauer *et al*. (2016) pointed at the importance of throughput. Their analysis demonstrated that citizens viewed policy processes more negatively if only business representatives were involved, but showed a higher procedure acceptance if a mixed set of interests – business and non‐business – was consulted. Nonetheless, we need to be careful with these results, as neither study tested for decision acceptance nor for the extent to which citizens were prepared to accept costly decisions in the field of climate policy. In addition, other studies bring nuance to this positive finding. For instance, a survey of stakeholder organizations conducted by Agné *et al*. (2015) did not find that consultations foster the legitimacy of global governance. Moreover, Dellmuth and Tallberg (2015) analyzed World Value Survey and European Values Study data from 26 countries and illustrated that the legitimacy of the United Nations (UN) is largely a matter of citizen confidence in its policy outputs and is not affected by consultation arrangements.

To summarize, the findings from extant empirical research contribute some skepticism to the presumed positive relations between consultations and legitimacy perceptions. Indeed, openness toward stakeholders is not necessarily a positive thing and may cause frustration among citizens. It could reinforce the perception among citizens that they have only limited influence or that the set of consulted stakeholders is one‐sided, because policymakers typically listen to a few important stakeholders, mostly business interests (Yackee 2006; Ulbig 2008). In addition, research indicates that consultations generally mobilize specialized business interests; therefore, consultation arrangements may stimulate capture and undermine the main reasons for agency policymaking (namely establishing Pareto‐optimal policy solutions), as it would result in suboptimal policies favoring regulated industries instead of serving the general interest (Stigler 1971; Posner 1974; Furlong & Kerwin 2005; Carpenter & Moss 2014). In short, the presumed positive relation between consultation arrangements, procedure, and decision acceptance cannot be taken for granted.

## 3 Hypotheses: legitimacy through consultations?

The empirical questions to be asked are whether consultations contribute to procedure and decision acceptance and, if so, which consultation arrangements have the strongest legitimizing potential? The considerations discussed above lead to our main hypothesis that decision‐making arrangements that involve stakeholder consultations are perceived as more acceptable than procedures without consultations. Importantly, however, consultation procedures can vary in terms of throughput legitimacy, more precisely the consultation format (*open* versus *closed*) and the types of interests that are represented (only business interests, only citizen interests, or both). These different interest constellations in consultations can shape how citizens and regulated companies evaluate procedures and decisions.

We distinguish between two consultation formats, open‐ versus closed‐access instruments, that vary in their degree of inclusiveness and in the extent to which stakeholders are able to deliberate among each other (Rasmussen & Toshkov 2013; Pedersen *et al*. 2015; Van Ballaert 2017; Arras & Beyers 2019; Beyers & Arras 2019). *Open‐access instruments* are usually online public consultations and have been explicitly promoted, both within the European and US contexts, as democratic consultation tools, given the relatively low cost of participation compared with face‐to‐face meetings with policymakers (e.g. West 2004; Furlong & Kerwin 2005; Quittkat 2011; Bunea 2017). Open consultations rely on the bottom‐up mobilization of stakeholders. When agencies wish to establish new policies or change policies, they launch an open call, inviting all interested stakeholders to submit their opinions and supply relevant information. Usually, such consultations and the submitted opinions are processed via website portals and may involve closed and/or open questions. In the final stage of the consultation, agencies publish a report summarizing all submissions and clarifying how the proposed policy was changed in view of the received opinions. Although a particular audience is sometimes addressed in consultation calls, in principle a wide and diverse array of stakeholders can participate. However, as the decision to become involved lies entirely with the stakeholders themselves, diverse stakeholder participation and/or deliberation among various stakeholders are not necessarily guaranteed or explicitly sought by the agency (Arras & Beyers 2019).

In contrast to open‐access instruments, *closed‐access consultations* can facilitate deliberation among stakeholders. Closed‐access instruments are advisory committees, which are usually permanent bodies within agencies. In these bodies, a limited number of stakeholders – interest groups, experts, or companies – hold seats over longer time periods (Moffitt 2010; Binderkrantz *et al*. 2015; Fraussen et al. 2014; Gornitzka & Sverdrup 2015; Rasmussen & Gross 2015; Arras & Beyers 2019). The key difference to open‐access instruments is that policymakers control who participates. Specific regulations may stipulate which actor types should be represented; although, agencies usually have some discretion regarding who may become a member of such committees. Given this format, the number of stakeholders in advisory committees is usually lower than the number involved in public consultations (Braun 2012). As agency leaders enjoy discretion regarding whom they consult, they can use this mechanism to diversify access and, in doing so, prevent excessive dependence on one stakeholder type. Hence, despite the smaller number of participants in committees (compared to open consultation), this consultation format has the potential to attract and facilitate deliberation among a more heterogeneous set of stakeholders (Arras & Beyers 2019).

Overall, we expect that both citizens and regulated companies perceive open consultation arrangements in which anyone who wishes to participate can do so as more acceptable than closed arrangements to which only a limited number of stakeholders are invited. Public consultations have the potential to attract many stakeholders and, as demonstrated by Rasmussen et al. 2014 and colleagues (2014), might be particularly effective in facilitating the mobilization of interests that reflect public opinion. Advisory committees are more exclusive than public consultations because they grant privileged access to a limited set of stakeholders. However, consultations through advisory committees may have some advantage compared to public consultations, as the former allows agencies to clearly communicate important information such as who was consulted, the diversity of the consulted stakeholders, and whether a consensual outcome was generated among these consultees.

While in general we expect that stakeholder involvement via advisory committees is perceived as less acceptable than public consultations, we expect differences between company leaders and citizens, depending on the configuration of interests represented on such committees.

From the perspective of *companies*, given the concentrated costs they face when confronted with regulatory policies, it is crucial that business interests are heard. Therefore, we expect that decisions and procedures will be perceived as more acceptable when stakeholders representing business interests belong to committees. This expectation fits within a cooptation logic, assuming that stakeholders who are closely involved during decision‐making are less likely to oppose the outcomes later on (Selznick 1949). This logic implies that companies will be less supportive of procedures that do not involve business interests; they might even perceive such procedures as just as (un)acceptable as decision‐making without any stakeholder consultation. *Citizens*, on the other hand, are more likely to exhibit a stronger allegiance to citizen groups, which often strive for stricter regulations (such as consumer groups or environmental NGOs) (Dür *et al*. 2015, p. 952; Dür & Mateo 2016). Citizens are therefore expected to prefer the inclusion of citizen groups in advisory committees and will be less supportive of procedures that involve only business interests. One might even expect that, for citizens, stakeholder involvement via committees that include business interests exclusively will be considered as equally (un)acceptable as no consultation at all.

Finally, consultation via committees that display a balance of interests, namely those that include both business and citizen interests, will be perceived as more acceptable than consultation via committees that include only one type of stakeholder. We expect this effect to work similarly for citizens and companies. Studies have indicated that regulated industries are better able than citizens' groups to mobilize resources over prolonged periods of time, are more actively involved in consultations, and have better access to agencies (Furlong & Kerwin 2005; Yackee & Yackee 2006; Fraussen et al. 2014; Yackee 2014; Chalmers 2015; Young & Pagliari 2015; Pagliari & Young 2016). From the perspective of citizens, a balanced representation may help to counter the assumed dominance of business interests in gaining access to and exerting influence on regulatory decision‐making (Bernauer & Gampfer 2013; Damonte *et al*. 2014; Bernauer *et al*. 2016). However, business interests may also prefer a balance of interests on committees, if representation of their views is guaranteed. Deliberation among stakeholders with different interests increases the overall legitimacy of regulations and prevents the perception that business dominates regulatory decision‐making.

Our hypotheses with respect to consultation arrangements can be summarized as follows:

Hypothesis 1 Decision‐making with any stakeholder consultation will be perceived as more acceptable (strong procedure and decision acceptance) than no consultation.

Hypothesis 2 Public consultations will generate the strongest procedure and decision acceptance, followed by advisory committees that display a balance of business and citizen groups.

Hypothesis 3a For citizens, committees that exclusively include citizen groups will be considered more acceptable than committees with only business representatives. Citizens might perceive the latter as equally (un)acceptable as no consultation at all.

Hypothesis 3b For company leaders, committees with exclusively business representatives will be considered more acceptable than committees with only citizen groups. Company leaders might perceive the latter as equally (un)acceptable as no consultation at all.

Table 1 summarizes how our expectations imply a ranking of the varying effects of consultation arrangements on procedure and decision acceptance.

**Table 1:** Consultation arrangements and the effect on procedure and decision acceptance

Individual citizens
No consultation ≤ committee only business groups < committee only citizen groups < committee business and citizen groups < public consultation
Companies
No consultation ≤ committee only citizen groups < committee only business groups < committee business and citizen groups < public consultation

These hypotheses presume a procedural logic, namely that consultation arrangements– open versus closed and types of stakeholders consulted – affect the extent to which citizens and regulated companies perceive decision‐making procedures and/or outcomes as legitimate. Citizens positively perceive procedures that consult stakeholders, and the more they perceive procedures as just, the more likely they are to accept decisions even if these decisions run counter their basic convictions. Procedures, not outcomes, drive legitimacy perceptions. This relation implies that *both* procedure and decision acceptance are positively affected by the consultation arrangements (Hypotheses 1, 2, 3a and 3b). Nonetheless, one could alternatively hypothesize that the acceptance of regulatory decisions is not substantially affected by the consultation arrangements. Instead, decision acceptance is primarily shaped by the overall predisposition toward government intervention (Grimmelikhuijsen & Meijer 2012; de Fine Licht 2014b). If so, individuals care much more about policy outputs, more precisely about the extent to which regulatory outcomes align with their basic policy views, than about the decision‐making procedure that was followed (see also Murphy *et al*. 2009; Gifford & Reisig 2019, p. 384). The pivotal importance of outputs makes the adopted consultation procedures a lesser concern. The implication is that the effects hypothesized in Table 1 only apply to procedure acceptance, but not to decision acceptance.

Few experimental studies control for how overall ideological orientations shape decision acceptance (Grimmelikhuijsen & Meijer 2012; Bernauer & Gampfer 2013; de Fine *et al*. 2014; de Fine Licht 2014a). However, it is generally presumed that citizens with a leftist political orientation are more prepared to accept government intervention that regulates and controls business activities (Hooghe *et al*. 2002; Prosser 2016). Conversely, business interests and citizens with a rightist orientation are less prone to accept interventionist regulatory outcomes. Therefore, more leftist and pro‐regulation‐oriented individuals will find more stringent environmental regulations more acceptable.

However, one might ask whether and under what conditions someone who is negatively predisposed toward government intervention is willing to accept an unfavorable outcome (for instance, more stringent regulation). Instead of positing a direct effect of left‐right orientation on decision acceptance, we hypothesize, in line with recent political science studies, that a decision that is in conflict with basic ideological orientations becomes more acceptable if individuals believe that the decision was made through an acceptable decision‐making procedure (Esaiasson 2010; Esaiasson *et al*. 2016, 2017; Strebel *et al*. 2018; for a similar argument in the procedural justice literature Murphy *et al*. 2009). Conversely, procedure acceptance matters less for decision acceptance when an outcome corresponds with an individual's political orientation. Or, a positive assessment of the decision‐making process, more precisely the consultation arrangement that was adopted, attenuates the experience of costly policies. Hence, we posit the following hypothesis:

Hypothesis 4 The more individuals judge a decision‐making procedure as acceptable, the greater the acceptance of decisions countering their political orientation.

## 4 Research design

To test whether and how consultations lead to procedure and decision acceptance, we conducted a survey experiment in which subjects were randomly presented with one of five newspaper articles about a fictitious regulation. Each article reported that the European Environment Agency (EEA) had proposed a new policy that would require organic packaging for all food products beginning in 2020 as part of a series of measures to reduce greenhouse gas emissions in the fight against climate change. Banning conventional plastics made from fossil fuels would decrease greenhouse gas emissions and reduce plastic pollution. True, the EEA has no formal competence to propose concrete regulations, nor does it organize formal consultations with organized interests. Yet, the EEA, whose main task is to disseminate expertise and knowledge, can assist the EU institutions in environmental policymaking and in this regard, it can formulate policy recommendations. Moreover, the EU regulation establishing the EEA does not prevent the EEA to consult with stakeholders (many EU agencies organize consultations, even if the framework legislation does not provide for consultation mechanisms; see Arras & Braun 2018). The main reason for designing an experiment with a fictitious case, which at the same time has real‐world resonance, was to limit the impact of unmeasured confounding factors such as ongoing political debates or media attention. The presentation of the newspaper article was followed by a short questionnaire. Although the vignette itself did not inform respondents about the fictitious nature of the case, we debriefed respondents by mentioning: (i) the fictitious nature of the case; and (ii) the fact that respondents were randomly assigned to one of the experimental stimuli (see Online Appendix). We also clarified the experimental design and respondents were given the opportunity to contact us for further questions.

Needless to say, environmental regulations can be highly contested and controversial, so that agencies are pressured to find a balance among the conflicting objectives put forward by various stakeholders. Biodegradable packaging, as well as transforming existing production processes, would incur considerable costs compared to maintaining the status quo (continued reliance on conventional plastics). The case may also imply costs for citizens, namely higher production costs may entail higher consumer prices and lower purchasing power. Of course, the regulation may generate benefits, namely a cleaner environment, but these are long‐term diffuse benefits. In short, the following factors characterizing the new regulation were kept constant in each vignette: (i) diffuse benefits (environmental protection); (ii) concentrated costs to the targeted industries; and (iii) costs to consumers in terms of purchasing power.

We designed five different versions of the newspaper article; each varied in the extent to which stakeholders were involved during the development of the regulation, while keeping other factors constant. This approach created the five experimental conditions listed in Table 2.

**Table 2:** Experimental conditions and assignment of citizens and companies (N)

		Citizens	Companies
Condition I	No consultation	436	49
Condition IIa	Committee only business groups	427	43
Condition IIb	Committee only citizen groups	421	40
Condition IIc	Committee business and citizen groups	435	48
Condition III	Public consultation	419	48

We operationalized procedure and decision acceptance using a slightly adapted version of questions developed by de Fine Licht (2011); de Fine Licht (2014a, 2014b); de Fine Licht *et al*. 2014). Procedure acceptance was measured by the following questions:

1. What do you think of how the decision was made?
2. How fairly do you think the decision was made?
3. How fairly do you think you were treated when the decision was made?

Regarding decision acceptance, we asked the following questions:

1. What do you think of the decision to require organic packaging for all foods?
2. How fair do you think the decision to require organic packaging for all foods is?
3. Given that the decision to require organic packaging for all foods involves costs, how acceptable do you find the decision?

The response format comprised seven‐point scales ranging from 1 (“very bad,” “very unfair,” or “very unacceptable”) to 7 (“very good,” “very fair,” or “very acceptable”) that were presented using a horizontal slider. Cronbach's alpha values for the procedure acceptance items are 0.90 (citizens) and 0.93 (companies); the values for the decision acceptance items are 0.90 (citizens) and 0.89 (companies). We combined the items in two additive indices ranging from 3 (minimum) to 21 (maximum), a procedure acceptance index based on the responses to the first three items and a decision acceptance index based on the responses to the next three items. Left–right orientation was measured by asking respondents to position themselves within a 0–10 range (using a horizontal slider), with 0 indicating the most leftist position and 10 the most rightist position. Table A1 in the Online Appendix presents an overview of the dependent and independent variables.

The experiment was conducted with two types of subjects: citizens and company leaders. For the citizens, we relied on a web‐survey panel of citizens established by the research group Media, Movements, and Politics at the University of Antwerp, Belgium. To determine whether the five vignettes worked as intended, we conducted a pilot study among 962 individuals who are part of the panel of which 376 individuals completed a pilot questionnaire. The respondents could provide us with feedback at the end of the questionnaire and their comments allowed us to improve the measurement instrument. We did not use these responses in the final analysis, due to small textual changes to the vignettes and some of the questions. The initial control vignette – measuring the no consultation condition – reported the regulatory outcome without providing information about whether or how stakeholders were involved. This led to numerous comments from respondents who received this vignette stating that they were not able to answer questions about stakeholder involvement due to a lack of information. Therefore, instead of including the control vignette, we opted for a more realistic approach, wherein the control condition explicitly informed respondents that neither business organizations nor consumer groups were involved during the decision‐making process (see also Esaiasson *et al*. 2017, p. 748; de Fine Licht 2014a). No substantial changes were made to the other four vignettes. We approached 4,685 individuals on the citizen panel, of whom 2,138 completed the final survey (response rate = 46%).

The list of companies was retrieved from the Belgian Crossroads Bank for Enterprises (CBE); the official register owned by the Belgian government that documents the official legal status of enterprises and organizations in Belgium. Given that the experimental conditions involved policymaking with respect to food packaging, we selected companies that are active in industries directly affected by such regulation. Based on 61 relevant NACE codes, we selected 5,148 companies in the food processing, packaging, retailing, and hospitality industries (for instance, restaurants, bars, food stores, and hotels).^1^ For each company, the CBE provides an e‐mail address (usually that of the manager, the CEO, or a spokesperson), and this was used to invite company leaders to participate in the experiment. In total, 228 company leaders took part in the experiment (response rate = 4.4%).

As our subjects were part of a self‐selected citizen panel, the results may not be representative of the broader population, which is part of a broader problem with the low external validity of experimental studies. To address these concerns, we implemented a wide range of robustness and manipulation checks. Details on key design features are reported in the Online Appendix; it concerns a clarification of sample representativeness and response rates, background information on the composition of the citizen panel, the overall set‐up of the experimental study, specific tests to reveal whether the experimental conditions worked as intended, and robustness checks with eight control variables testing whether the random assignment of subjects to the five conditions did not result in confounding factors.

## 5 Results

To test the hypotheses, we proceeded in two steps. First, we tested Hypothesis 1, 2, 3a and 3b by running four analyses of variance (ANOVA) tests summarizing how the five experimental treatments affect procedure and decision acceptance (Table 3). As the randomization did not work well for the variable “general attitude towards consultations,” we tested additional models with this control variable (and left–right orientation); these models are reported in the Online Appendix (Table A3 and Table A4). Second, Table 4 tests Hypothesis 4 on how procedure acceptance moderates the impact of political orientation on decision acceptance. To simplify the interpretation and comparison of the regression coefficients, we scaled dependent and independent variables on a [0–1]‐interval, which eases the comparison of the magnitude of the unstandardized coefficients. Levene‐ and White‐tests demonstrated that the assumption of homogeneity of variances was not met in Model I (Table 3) and Model IV–VIII (Table 4). Therefore, all models were re‐estimated with heteroscedasticity‐consistent standard errors (or Huber‐White standard errors) and a Welch‐test; these analyses show almost identical results compared to the findings reported in this paper. Details are available in the Online Appendix.

**Table 3:** The effect of consultation arrangement on procedure and decision acceptance (Hypothesis 1, 2, 3a and 3b)

	Procedure		Decision	
	Citizen	Company	Citizen	Company
	Model I	Model II	Model III	Model IV
Intercept	0.35 (0.01)***	0.25 (0.03)***	0.71 (0.01)***	0.60 (0.04)***
Stakeholder consultation:				
I. No consultation (=ref.)	—	—	—	—
IIa. Closed only business	0.01 (0.01)	0.09 (0.02)	−0.09 (0.02)	−0.00 (0.06)
IIb. Closed only consumers	0.08 (0.01)***	0.05 (0.05)	−0.00 (0.02)	−0.02 (0.06)
IIc. Closed business and consumers	0.17 (0.02)***	0.19 (0.05)***	0.00 (0.02)	0.01 (0.06)
III. Public consultation	0.09 (0.02)***	0.14 (0.05)**	−0.02 (0.02)	0.06 (0.06)
R^2^	0.07	0.08	0.00	0.01
F	39.10	4.79	0.74	0.56
Df	4	4	4	4
N	2,138	219	2,138	219

*P < 0.05; **P < 0.01; ***P < 0.001.

All variables are scores to range from 0 to 1; unstandardized regression coefficients; standard errors in parentheses

**Table 4:** The interaction effect of political orientation and procedure acceptance on decision acceptance (Hypothesis 4)

	Individual citizens		Company leaders	
	Non‐interactive Model V	Interactive Model VI	Non‐interactive Model VII	Interactive Model VIII
Intercept	0.60 (0.01)***	0.68 (0.02)***	0.55 (0.06)***	0.54 (0.09)***
Procedure acceptance (PA)	0.51 (0.02)***	0.33 (0.04)***	0.41 (0.07)***	0.45 (0.21)*
Left–right	−0.22 (0.02)***	−0.36 (0.04)***	−0.14 (0.08)	−0.12 (0.14)
Procedure PA × left–right		0.36 (0.07)***		−0.07 (0.35)
R^2^	0.29	0.29	0.15	0.15
F	429.66	297.26	18.65	12.39
Df	2	3	2	3
N	2,138	2,138	213	213

*P < 0.05; **P < 0.01; ***P < 0.001.

All variables are scores to range from 0 to 1; unstandardized regression coefficients; standard errors in parentheses.

In Model I (Table 3), we observe that how stakeholders are consulted significantly affects the extent to which citizens accept the decision‐making procedure. The control condition of no stakeholder consultation is considered as less acceptable than arrangements with consultations, which confirms Hypothesis 1 for citizens. As expected, the insignificant coefficient for consultation via an advisory committee in which only business interests are represented indicates that such closed consultations are not significantly different from the no consultation condition(F_(1,2,138)_ = 0.53, *P* = 0.4679). Individuals find committees with only citizen groups more acceptable than consultations with only business stakeholders (F_(1,2,138)_ = 17.44, *P* = 0.0001, *ω* ^2^ = 0.01, CI_95%_ = [0.00, 0.02]). This supports Hypothesis 3a, for citizens, committees with only citizen groups are more acceptable than committees with only business representatives; the latter are seen as equally unacceptable as procedures without consultations. The most accepted arrangement is, contrary to what we proposed with Hypothesis 2, a closed consultation rather than a public consultation. More precisely, advisory committees with *both* business and citizen groups are perceived more positively than open consultations. The positive impact of this condition on procedure acceptance is significantly higher than the effect of no consultations (F_(1,2,138)_ = 119.22, *P* = 0.0001, *ω* ^2^ = 0.05, CI_95%_ = [0.04,0.07]), public consultations (F_(1,2,138)_ = 22.25, *P* = 0.0001, *ω* ^2^ = 0.01, CI_95%_ = [0.00,0.02]), closed consultations involving only consumer groups (F_(1,2,138)_ = 34.87, *P* = 0.0001, *ω* ^2^ = 0.02, CI_95%_ = [0.01,0.03]), and closed consultations with only business stakeholders (F_(1,2,138)_ = 102.74, *P* = 0.0001, *ω* ^2^ = 0.05, CI_95%_ = [0.03,0.06]). Concretely, the predicted procedure acceptance for these mixed closed arrangements is 0.51(CI_95%_ = [0.49, 0.54]), substantially higher than the acceptance of the no consultation condition (*ŷ* = 0.35, CI_95%_ = [0.32, 0.37]), the open consultation condition (*ŷ* = 0.44, CI_95%_ = [0.42, 0.46]), and consultations with only business (*ŷ* = 0.35, CI_95%_ = [0.32, 0.37]) or consumer representatives only (*ŷ* = 0.42, CI_95%_ = [0.40, 0.44]).

Model II (Table 3) presents the results for company leaders. As expected, procedures with consultations are considered more acceptable than the no consultation condition (Hypothesis 1). While arrangements with only business stakeholders are not considered as more acceptable than those with only consumer interests (F_(1,219)_ = 0.92, *P* = 0.3383), similar to citizens, the most acceptable procedure is a closed arrangement where *both* business interests and citizen groups are represented. The acceptance of this arrangement is significantly higher than the no consultation condition (F_(1,219)_ = 15.72, *P* = 0.0001, *ω* ^2^ = 0.06, CI_95%_ = [0.02, 0.14]) and closed consultation with only consumer stakeholders (F_(1,219)_ = 8.10, *P* = 0.0049, *ω* ^2^ = 0.03, CI_95%_ = [0.00, 0.10]). Concretely, the predicted procedure acceptance is 0.44 (CI_95%_ = [0.37, 0.50]) for mixed arrangements, which is higher than the predicted outcome for the no consultation condition (*ŷ* = 0.25, CI_95%_ = [0.19, 0.32]) and the only consumers condition (*ŷ* = 0.30, CI_95%_ = [0.23, 0.37]). It should be noted that the predicted effect of a mixed arrangement (*ŷ* = 0.44) does not differ significantly from the open consultations (*ŷ* = 0.39; F_(1,219)_ = 1.12, *P* = 0.2903) or consultations with only business groups (*ŷ* = 0.35, F_(1,219)_ = 3.54, *P* = 0.0611). Hence, Hypotheses 2 and 3b are partially confirmed. More precisely, company leaders view a consultation with only citizen groups as equally (un)acceptable as the no consultation condition (F_(1,219)_ = 0.92, *P* = 0.3377) (Hypothesis 4), but they prefer mixed arrangements (Hypothesis 2) over other arrangements (including those with only business representatives).

Models III and IV (Table 3) demonstrate that, for both citizens and company leaders, decision acceptance is not substantially affected by the consultation arrangement. Hence, the expected effects (Hypotheses 1, 2, 3a and 3b) apply for procedure acceptance, but not for decision acceptance. Table A4 in the Online Appendix adds two control variables – left–right and general attitude toward consultations – and demonstrates a significant effect of political orientation. In addition to the consultation arrangement, political orientation also significantly affects procedure acceptance, although the magnitude of this effect is less substantial. The more leftist, the more acceptable an individual finds the adopted decision‐making procedure. Moreover, compared to procedure acceptance, the effect of political orientation is greater for decision acceptance. More precisely, if citizens move from the most leftist (0) to the most rightist position (1), their predicted decision acceptance decreases on average by 0.23 (SE_B_ = 0.02, *P* < 0.0001, CI_95%_ = [−0.27, −0.19]); a similar effect is observed for company leaders (B = −0.21, SE_B_ = 0.09, *P* = 0.0197, CI_95%_ = [−0.39, −0.03]). This result is also reflected when comparing the descriptive results in Table A1; average citizens are, compared to company leaders, more prone to accept the decision and the procedure. Moreover, average company leaders are positioned more to the right, which corresponds with their lower propensity to accept the proposed regulation. In line with the alternative expectation, while procedure acceptance is affected by the consultation arrangement, decision acceptance remains unaffected by the consultation arrangement. In addition, a right‐wing orientation lowers the propensity to accept the consultation arrangement, but it especially suppresses the propensity to accept a policy decision entailing a stricter and potentially costly regulation on food packaging.

We hypothesized that individuals might care more about the extent to which regulatory outcomes align with their overall predisposition toward government intervention, than about inputs or the quality of the adopted consultation arrangement (Grimmelikhuijsen & Meijer 2012; de Fine Licht 2014b). Can we conclude that decision‐making procedures (and for instance the involvement of stakeholders)are of a lesser concern? Under what conditions are citizens or company leaders, including those negatively predisposed toward government intervention, prepared to accept unfavorable policies (for instance, more stringent regulations)? Hypothesis 4 states that decisions that conflict with basic political convictions may become more acceptable if individuals believe that the decision was made through procedures they consider as more acceptable.

Model V (citizens) and Model VII (company leaders) in Table 4 demonstrate that procedure acceptance is positively related to decision acceptance; more precisely, the difference in the predicted decision acceptance between the lowest‐scoring (0) and the highest‐scoring respondent (1) on the procedure acceptance scale is 0.50 (SE_B_ = 0.02, *P* < 0.0001, CI_95%_ = [0.47, 0.54]) for citizens and 0.41 (SE_B_ = 0.07, *P* < 0.0001, CI_95%_ = [0.27, 0.55])for companies. For citizens, we still observe a significant impact of political orientation, but for companies we find that the average change in decision acceptance due to political orientation is insignificant. Model VIII rejects Hypothesis 4 for company leaders, namely that procedure acceptance moderates the impact of left–right orientation. However, similar as for citizens, the relation between the consultation arrangement and decision acceptance runs indirectly via procedure acceptance. The predicted values clearly illustrate the significant relation between procedure acceptance and decision acceptance; the average predicted decision acceptance equals 0.61 (CI_95%_ = [0.55, 0.66]) but increases to 0.88 (CI_95%_ = [0.78, 0.98]) at the highest level of procedure acceptance, while it is 0.47 (CI_95%_ = [0.41, 0.54]) at the lowest level.

**Figure 1:**
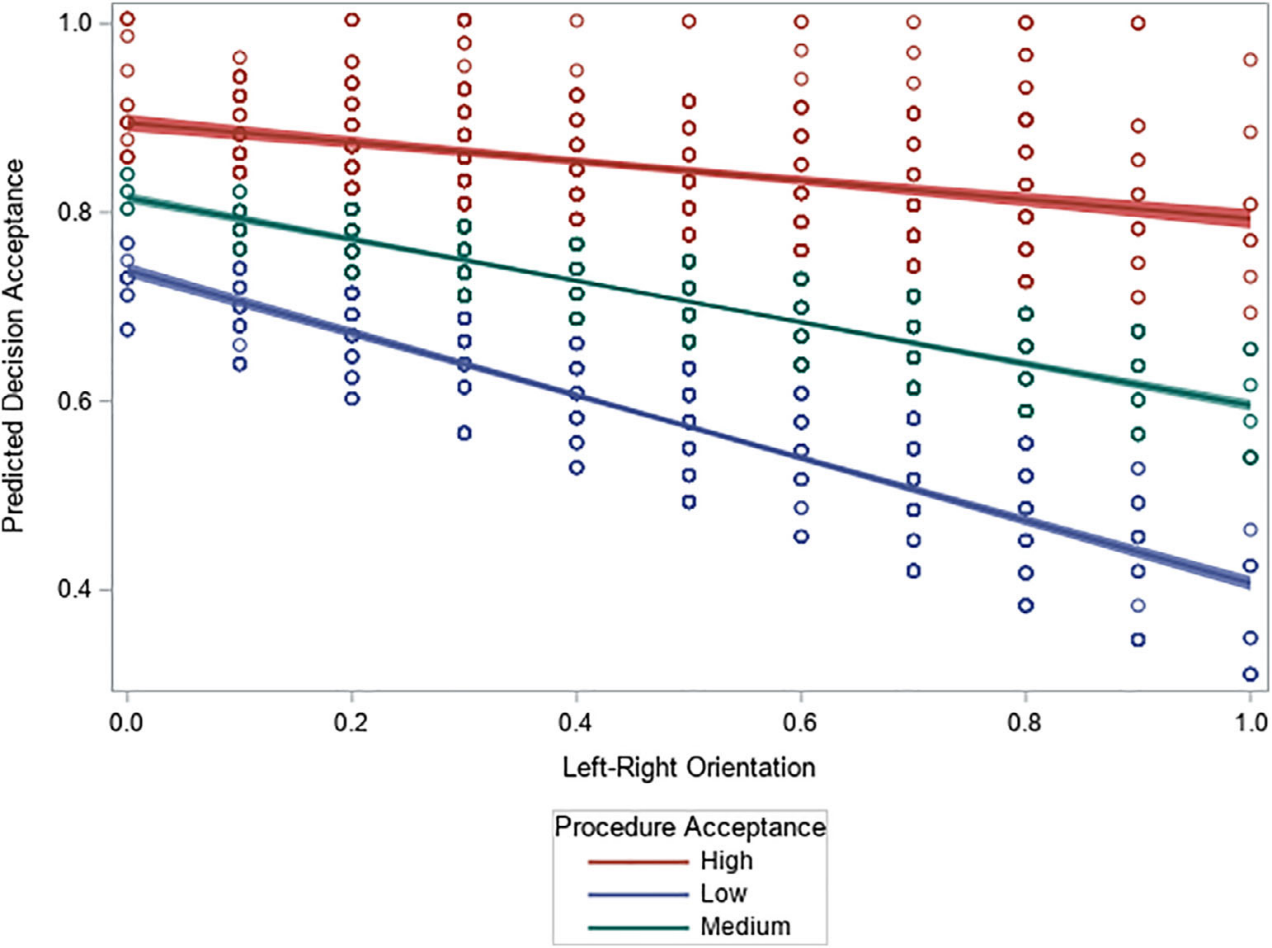
Predicted decision acceptance by procedure acceptance and left–right orientation (Model IV, 95% confidence interval).

Model VI confirms Hypothesis 4 by indicating a significant interaction between procedure acceptance and left–right orientation. The impact of political orientation clearly depends on the extent to which citizens judge a consultation arrangement in a positive way. Figure 1 presents the predicted decision acceptance (y‐axis) as a function of left–right orientation (x‐axis) for three levels of procedure acceptance (red slope = 33.3% highest acceptance; green slope = 33.3% medium acceptance; blue slope = 33.3% lowest acceptance). Significance tests reported in the Online Appendix (Table A5) demonstrate that, at varying levels of political orientation, the slopes are statistically different from each other. Although each slope indicates a lower decision acceptance as someone becomes more rightist, the overall decision acceptance is higher for individuals with higher procedure acceptance. Importantly, it is especially among respondents with low procedure acceptance that we observe a steep decline in the predicted decision acceptance (blue slope; decrease from *ŷ* = 0.72, CI_95%_ = [0.69, 0.75] to *ŷ* = 0.36, CI_95%_ = [0.33, 0.40]) when the respondents become more right‐wing. Similarly, predicted decision acceptance declines much less (red slope; decrease from *ŷ* = 0.91, CI_95%_ = [0.88, 0.95] to *ŷ* = 0.80, CI_95%_ = [0.77, 0.84]) when somebody is right‐wing and demonstrates a high level of procedure acceptance. Outcomes that are less congruent with an individual's ideological view are more readily accepted if consultation arrangements are judged positively and procedure acceptance matters less for decision acceptance when an outcome corresponds with the individual's political orientation. Hence, trust in the decision‐making procedure and in the related consultation arrangement attenuates the experience of potentially controversial policies.

## 6 Discussion and conclusion

We set out to analyze the relation between consultations and legitimacy in the specific context of an environmental regulation. We hypothesized that regulatory decision‐making is perceived as more legitimate when procedures facilitate stakeholder input and/or guarantee throughput legitimacy. With respect to the consultation arrangements, we distinguished between open and closed procedures, namely public consultations in which anyone who wishes to participate can do so versus advisory committees with only a limited set of stakeholders that enjoy access to the decision‐making process. Additionally, we made a distinction between heterogenous advisory committees (with business and citizen interests) and more homogeneous advisory committees (only business or only citizen interests). Alternatively, we expected that how decisions are made would not matter for decision acceptance, if regulatory outcomes are in line with an individual's political orientation.

We tested these expectations via a survey experiment among citizens and companies in Belgium. Our main experimental result is that consultations can indeed improve procedure acceptance, but have no direct effect on decision acceptance. Individuals who agree with decisions may care less about specific consultation arrangements. Therefore, whatever the decision‐making procedure, they will, because they agree with the regulatory outcome, demonstrate greater procedure acceptance. Hence, we should be cautious with qualifying the association between procedure and decision acceptance as a causal relationship. It could be that most of our respondents are so favorable about the outcome that they simply care less about consultation arrangements. Although the uncontested nature of climate change in Belgium is reflected in the high decision acceptance among both citizens and company leaders, the acceptance values still indicate substantial variation. Whereas the average decision acceptance is, measured on a 0‐1 scale, 0.61 for companies and 0.71 for citizens, 40% of the citizens and 63% of the company leaders did not approve (scored 0.33 or lower on the decision acceptance scale) or only weakly supported (scored lower, namely between 0.33 and 0.66 on the decision acceptance scale) the regulation. Hence, different results might be obtained in political systems where this topic is more divisive (for instance, climate change policies in the US) or in areas about which citizens hold strong moral convictions (for instance, immigration and border control, see de Fine Licht 2014b; Earle & Siegrist 2008).

Some might conclude that procedure and decision acceptance are so closely related that they in fact are components representing one single underlying trait (Jackson 2018, pp. 152–153). However, a significant statistical correlation does not confirm conceptual equivalence. Instead, the differential effect of the consultation arrangement on the two dependent variables (Table 3) suggests that procedure and decision acceptance are two distinct, albeit related, concepts. True, Table 3 does not demonstrate a direct effect of the consultation arrangement on decision acceptance, but it confirms that the consultation arrangement directly improves procedure acceptance. The next analysis (Table 4) demonstrates that procedure acceptance is positively related to decision acceptance and that, among citizens, procedure acceptance moderates the impact of political orientation on decision acceptance. Procedure acceptance makes it for individuals with a less pro‐regulatory disposition easier to accept a potentially costly regulation. This interaction effect, and relatedly the efforts of agencies to improve procedure acceptance, are not irrelevant, as it may clarify why, with respect to this issue, rightist individuals accept a regulatory outcome. This confirms earlier findings in both the political science (Esaiasson 2010; Esaiasson *et al*. 2016, 2017; Strebel *et al*. 2018) and procedural justice literature (Murphy *et al*. 2009) stating that procedural legitimacy or procedural justice is more important for decision acceptance or compliance among individuals who are confronted with regulations that conflict with their basic beliefs. Conversely, the moderation effect is much weaker for individuals who perceive a policy as legitimate; these individuals will accept regulations irrespective of the nature of the decision‐making process.

We admit that the significant interaction effects of political orientation and procedure acceptance do not demonstrate a causal relationship. However, theoretically it is plausible that basic political orientations (such as left–right) are more deeply ingrained than and precede the adoption of policy positions with respect to concrete issues (Sabatier 1988; Yee 1996). Ideological predispositions do not abruptly change when individuals are confronted with specific political events (such as concrete regulatory decisions). Rather, it is plausible to presume that an individual's positioning with respect to concrete policy issues is shaped by his or her basic political orientations.

We can draw three other relevant conclusions. First, although open consultations have been promoted as a tool that can be used to reach a wider public and thereby render decision‐making more inclusive, thus improving the legitimacy of regulatory governance, our results suggest that open consultations are not a miracle solution to increase procedural legitimacy. Second, concerning consultations through advisory committees, where the set of invited stakeholders is more identifiable (compared to public consultations), individuals are sensitive to the configurations in which their interests as consumers or as company leaders are not represented. Specifically, company leaders consider committees that include only citizen groups equivalent to the “no consultation” condition, while citizens perceive no difference between committees that include exclusively business interests and the “no consultation” condition. Third, consultations characterized by a diverse set of interests – citizen groups *and* business interests – have the strongest potential to improve legitimacy perceptions by both citizens and companies (Bernauer *et al*. 2016, pp. 441–444). These results point to the crucial importance of throughput legitimacy; especially preventing the dominance of one particular stakeholder type and the importance of facilitating interactions between stakeholders with different views, eventually leading to better regulatory outcomes (de Fine Licht *et al*. 2014).

While we designed our study to maximize theoretical validity and while our results are in line with those of several recent studies (Esaiasson 2010; Bernauer & Gampfer 2013; Bernauer *et al*. 2016; Esaiasson *et al*. 2016, 2017; Arnesen 2017; Porumbescu & Grimmelikhuijsen 2018; Strebel *et al*. 2018), we should remain cautious about generalizing to other institutional contexts. The study implemented among Belgian subjects might have affected the research outcome. The experimental conditions referred to instances in which stakeholders are actively involved in decision‐making processes; these practices resemble corporatist forms of policymaking with which Belgians are quite familiar. The consensual nature of Belgian politics might make respondents more sensitive, perhaps in a positive way, to negotiated outcomes (see also Esaiasson *et al*. 2017). Hence, it would be worthwhile to conduct similar experiments among individuals who are socialized in more pluralist contexts (for instance, the UK or the US).

Future research should examine the relation between consultations and legitimacy across different policies and institutional contexts, including policies that aim to deregulate the status quo, and could include more controversial issues in other areas. Another useful topic for follow‐up research concerns the role of information and policy complexity; we can imagine that highly complex cases require different and more elaborate consultation arrangements. For instance, our tests presumed that agencies use one single consultation format, while in reality agencies combine open and closed consultations with informal interactions and often vary the use of consultation instruments during various stages of the policy process (West 2004). Therefore, relevant avenues for future research concern how combinations of consultation instruments affect legitimacy perceptions.

Finally, citizens' perceptions of consultations may not reflect the actual extent of stakeholder input or the type of consultations, but rather result from cues reported in the media (de Fine Licht 2011; de Fine Licht *et al*. 2014; de Fine Licht 2014a). Conventional laboratory experiments simulating actual decision‐making procedures, rather than survey experiments, may shed more light on how the actual extent or the consultation formats would affect procedure and decision acceptance. Nonetheless, our results point to the importance of how policymakers themselves communicate about and explain to stakeholders and the general public the policymaking process and its outcome (Esaiasson *et al*. 2017, p. 742; Porumbescu & Grimmelikhuijsen 2018). Ultimately, citizens and companies need to be informed about policies and how these were established, and the news media play a pivotal role in that regard. Although a possible disturbing implication of our analysis is that how policymakers and the news media report on actual policy processes could make citizens vulnerable to manipulation, a positive result is that consultations, if these facilitate deliberative interactions with and among a diverse array of stakeholders, may contribute positively to legitimacy perceptions among citizens and regulated companies.

## Data Availability Statement

Replication materials are available in the dataverse‐account of the corresponding author at https://dataverse.harvard.edu/.

## Supporting information


Appendix

## Endnotes

1 NACE stands for the French ‘Nomenclature Statistique des Activités économiques dans la Communauté Européenne’, which is equivalent to the United Nations ‘International Standard Industrial Classification of All Economic Activities'ISIC classification system (ISIC). The list of 61 NACE‐codes can be obtained from the authors.

## References

[rego12323-bib-0001] Agné H, Dellmuth LM, Tallberg J (2015) Does Stakeholder Involvement Foster Democratic Legitimacy in International Organizations? An Empirical Assessment of a Normative Theory. Review of International Organizations 10, 465–488.

[rego12323-bib-0401] Arras S, Beyers J (2019) Access to European Union agencies. Usual suspects or balanced interest representation in open and closed consultations? Journal of Common Market Studies 10.1111/jcms.12991. PMC7386941 32742020

[rego12323-bib-0402] Arras S, Braun C (2018) Stakeholders wanted! Why and how European Union agencies involve non‐state stakeholders. Journal of European Public Policy 25, 1257–1275.

[rego12323-bib-0004] Arnesen S (2017) Legitimacy from Decision‐Making Influence and Outcome Favourability: Results from General Population Survey Experiments. Political Studies 65, 146–161.

[rego12323-bib-0006] Bernauer T, Gampfer R (2013) Effects of Civil Society Involvement on Popular Legitimacy of Global Environmental Governance. Global Environmental Change 23, 439–449.

[rego12323-bib-0007] Bernauer T, Gampfer R, Meng T, Su Y‐S (2016) Could more Civil Society Involvement Increase Public Support for Climate Policy‐Making? Evidence from a Survey Experiment in China. Global Environmental Change 40, 1–12.

[rego12323-bib-0404] Beyers J, Arras S (2019) Who feeds information to regulators? Stakeholder diversity in European Union regulatory agency consultations. Journal of Public Policy 10.1017/S0143814X19000126.

[rego12323-bib-0008] Binderkrantz AS, Christiansen PM, Pedersen HH (2015) Interest Group Access to the Bureaucracy, Parliament, and the Media. Governance 28, 95–112.

[rego12323-bib-0009] Blom‐Hansen J, Morton R, Serritzlew S (2015) Experiments in Public Management Research. International Public Management Journal 18, 151–170.

[rego12323-bib-0010] Borrás S, Koutalakis C, Wendler F (2007) European Agencies and Input Legitimacy: EFSA, EMeA and EPO in the Post‐Delegation Phase. Journal of European Integration 29, 583–600.

[rego12323-bib-0012] Braun C (2012) The Captive or the Broker? Explaining Public Agency‐Interest Group Interactions. Governance 25, 291–314.

[rego12323-bib-0013] Bunea A (2017) Designing Stakeholder Consultations: Reinforcing or Alleviating Bias in the European Union System of Governance? European Journal of Political Research 54, 46–69.

[rego12323-bib-0014] Busuioc M (2009) Accountability, Control and Independence: The Case of European Agencies. European Law Journal 15, 599–615.

[rego12323-bib-0017] Carpenter DP, Moss DA (2014) Preventing Regulatory Capture: Special Interest Influence and How to Limit it. Cambridge University Press, Cambridge.

[rego12323-bib-0018] Chalmers AW (2015) Financial Industry Mobilisation and Securities Markets Regulation in Europe. European Journal of Political Research 54, 482–501.

[rego12323-bib-0019] Damonte A, Dunlop CA, Radaelli CM (2014) Controlling Bureaucracies with Fire Alarms: Policy Instruments and Cross‐Country Patterns. Journal of European Public Policy 21, 1330–1349.

[rego12323-bib-0020] Dellmuth LM, Tallberg J (2015) The Social Legitimacy of International Organisations: Interest Representation, Institutional Performance, and Confidence Extrapolation in the United Nations. Review of International Studies 41, 451–475.

[rego12323-bib-0021] Druckman JN, Green DP, Kuklinski JH, Lupia A (2011) Cambridge Handbook of Experimental Political Science. Cambridge University Press, Cambridge.

[rego12323-bib-0022] Dür A, Mateo G (2016) Insiders Versus Outsiders: Interest Group Politics in Multilevel Europe. Oxford University Press, Oxford.

[rego12323-bib-0023] Dür A, Bernhagen P, Marshall D (2015) Interest Group Success in the European Union: When (and why) Does Business Lose? Comparative Political Studies 48, 951–983.

[rego12323-bib-0024] Earle TC, Siegrist M (2008) On the Relation between Trust and Fairness in Environmental Risk Management. Risk Analysis 28, 1395–1414. 18631299 10.1111/j.1539-6924.2008.01091.x

[rego12323-bib-0025] Egeberg M, Trondal J (2017) Researching European Union Agencies: What Have We Learnt (and Where Do We Go from Here)? Journal of Common Market Studies 55, 675–690.

[rego12323-bib-0026] Esaiasson P (2010) Will Citizens Take no for an Answer? What Government Officials Can Do to Enhance Decision Acceptance. European Political Science Review 2, 351–371.

[rego12323-bib-0027] Esaiasson P, Gilljam M, Persson M (2012) Which Decision‐Making Arrangements Generate the Strongest Legitimacy Beliefs? Evidence from a Randomised Field Experiment. European Journal of Political Research 51, 785–808.

[rego12323-bib-0028] Esaiasson P, Persson M, Gilens M, Lindholm T (2016) Reconsidering the Role of Procedures for Decision Acceptance. British Journal of Political Science 49, 291–314. 10.1017/S0007123416000508

[rego12323-bib-0029] Esaiasson P, Gilljam M, Persson M (2017) Responsiveness beyond Policy Satisfaction: Does It Matter to Citizens? Comparative Political Studies 50, 739–765.

[rego12323-bib-0030] de Fine Licht J (2011) Do We Really Want to Know? The Potentially Negative Effect of Transparency in Decision Making on Perceived Legitimacy. Scandinavian Political Studies 34, 183–201.

[rego12323-bib-0031] de Fine Licht J (2014a) Transparency Actually: How Transparency Affects Public Perceptions of Political Decision‐Making. European Political Science Review 6, 309–330.

[rego12323-bib-0032] de Fine Licht J (2014b) Policy Area as a Potential Moderator of Transparency Effects: An Experiment. Public Administration Review 74, 361–371.

[rego12323-bib-0033] de Fine Licht J, Naurin D, Esaiasson P, Gilljam M (2014) When Does Transparency Generate Legitimacy? Experimenting on a Context‐Bound Relationship. Governance 27, 111–134.

[rego12323-bib-0034] Font N, Pérez Durán I (2016) The European Parliament Oversight of EU Agencies through Written Questions. Journal of European Public Policy 23, 1349–1366.

[rego12323-bib-0409] Fraussen B, Beyers J, Donas T (2014) The Expanding Core and Varying Degrees of Insiderness Institutionalized Interest Group Involvement Through Advisory Councils. Political Studies 63, 569–588.

[rego12323-bib-0035] Furlong SR (1997) Interest Group Influence on Rule Making. Administration & Society 29, 325–347.

[rego12323-bib-0036] Furlong SR, Kerwin CM (2005) Interest Group Participation in Rule Making: A Decade of Change. Journal of Public Administration Research and Theory 15, 353–370.

[rego12323-bib-0037] Gifford FE, Reisig MD (2019) A Multidimensional Model of Legal Cynicism. Law and Human Behavior 43(4), 383–396. 30958019 10.1037/lhb0000330

[rego12323-bib-0038] Gornitzka Å, Sverdrup U (2015) Societal Inclusion in Expert Venues: Participation of Interest Groups and Business in the European Commission Expert Groups. Politics & Governance 3, 151–165.

[rego12323-bib-0039] Grimmelikhuijsen SG (2012) Linking Transparency, Knowledge and Citizen Trust in Government: An Experiment. International Review of Administrative Sciences 78, 50–73.

[rego12323-bib-0040] Grimmelikhuijsen SG, Meijer AJ (2012) Effects of Transparency on the Perceived Trustworthiness of a Government Organization: Evidence from an Online Experiment. Journal of Public Administration Research and Theory 24, 137–157.

[rego12323-bib-0041] Grimmelikhuijsen SG, Porumbescu GA, Hong B, Tobin T (2013) The Effect of Transparency on Trust in Government: A Cross‐National Comparative Experiment. Public Administration Review 73, 575–586.

[rego12323-bib-0042] Grimmelikhuijsen SG, Herkes F, Leistikow I, Verkroost J, de Vries F, Zijlstra WG (2019) Can Decision Transparency Increase Citizen Trust in Regulatory Agencies? Evidence from a Representative Survey Experiment. Regulation & Governance . 10.1111/rego.12278.

[rego12323-bib-0043] Hooghe L, Marks G, Wilson CJ (2002) Does Left/Right Structure Party Positions on European Integration? Comparative Political Studies 35, 965–989.

[rego12323-bib-0044] Jackson J (2018) Norms, Normativity, and the Legitimacy of Justice Institutions: International Perspectives. Annual Review of Law and Social Science 14, 145–165.

[rego12323-bib-0045] Kelemen DR (2002) The Politics of “Eurocratic” Structure and the New European Agencies. West European Politics 25, 93–118.

[rego12323-bib-0046] Levi‐Faur D (2005) The Global Diffusion of Regulatory Capitalism. The ANNALS of the American Academy of Political and Social Science 598, 12–32.

[rego12323-bib-0047] Majone G (1999) The Regulatory State and its Legitimacy Problems. West European Politics 22, 1–24.

[rego12323-bib-0049] McCubbins MD, Schwartz T (1984) Congressional Oversight Overlooked: Police Patrols Versus Fire Alarms. American Journal of Political Science 28, 165–179.

[rego12323-bib-0050] Moffitt SF (2010) Promoting Agency Reputation through Public Advice: Advisory Committee Use in the FDA. Journal of Politics 72, 880–893.

[rego12323-bib-0052] Murphy K, Tyler TR, Curtis A (2009) Nurturing Regulatory Compliance: Is Procedural Justice Effective When People Question the Legitimacy of the Law? Regulation & Governance 3, 1–26.

[rego12323-bib-0053] Pagliari S, Young KL (2016, 14) The Interest Ecology of Financial Regulation: Interest Group Plurality in the Design of Financial Regulatory Policies. Socio‐Economic Review 14, 309–337.

[rego12323-bib-0054] Pedersen HH, Halpin DR, Rasmussen A (2015) Who Gives Evidence to Parliamentary Committees ? A Comparative Investigation of Parliamentary Committees and their Constituencies. Journal of Legislative Studies 21, 408–427.

[rego12323-bib-0055] Porumbescu GA, Grimmelikhuijsen SG (2018) Linking Decision‐Making Procedures to Decision Acceptance and Citizen Voice: Evidence from Two Studies. American Review of Public Administration 48, 902–914.

[rego12323-bib-0056] Posner RA (1974) Theories of Economic Regulation. Bell Journal of Economics and Management Science 5, 335–358.

[rego12323-bib-0057] Prosser C (2016) Dimensionality, Ideology and Party Positions towards European Integration. West European Politics 39, 731–754.

[rego12323-bib-0058] Quittkat C (2011) The European Commission's Online Consultations: A Success Story? Journal of Common Market Studies 49, 653–674.

[rego12323-bib-0059] Quittkat C, Kohler‐Koch B (2013) Involving Civil Society in EU Governance: The Consultation Regime of the European Commission. In: Kohler‐Koch B, Quittkat C (eds) De‐Mystification of Participatory Democracy, pp. 41–61. Oxford, Oxford University Press.

[rego12323-bib-0061] Rasmussen A, Carroll BJ (2014) Determinants of Upper‐Class Dominance in the Heavenly Chorus: Lessons from European Union Online Consultations. British Journal of Political Science 44, 445–459.

[rego12323-bib-0062] Rasmussen A, Gross V (2015) Biased Access? Exploring Selection to Advisory Committees. European Political Science Review 7, 343–372.

[rego12323-bib-0063] Rasmussen A, Toshkov D (2013) The Effect of Stakeholder Involvement on Legislative Duration: Consultation of External Actors and Legislative Duration in the European Union. European Union Politics 14, 366–387.

[rego12323-bib-0064] Rasmussen A, Carroll BJ, Lowery D (2014) Representatives of the Public? Public Opinion and Interest Group Activity. European Journal of Political Research 53, 250–268.

[rego12323-bib-0065] Rittberger B, Wonka A (2011) Introduction: Agency Governance in the European Union. Journal of European Public Policy 18, 780–789.

[rego12323-bib-0066] Sabatier PA (1988) An Advocacy Coalition Framework of Policy Change and the Role of Policy‐Oriented Learning Therein. Policy Sciences 21, 129–168.

[rego12323-bib-0067] Scharpf FW (1999) Governing in Europe: Effective and Democratic? Oxford University Press, New York.

[rego12323-bib-0068] Schmidt VA (2013) Democracy and Legitimacy in the European Union Revisited: Input, Output and 'Throughput'. Political Studies 61, 2–22.

[rego12323-bib-0069] Selznick P (1949) TVA and the Grassroots. A Study in the Sociology of Formal Organizations. University of California Press, Berkeley.

[rego12323-bib-0070] Stigler GJ (1971) The Theory of Economic Regulation. Bell Journal of Economics and Management Science 2, 3–21.

[rego12323-bib-0071] Strebel MA, Kübler D, Marcinkowski F (2018) The Importance of Input and Output Legitimacy in Democratic Governance: Evidence from a Population‐Based Survey Experiment in Four West European Countries. European Journal of Political Research 58, 488–513.

[rego12323-bib-0072] Thibaut J, Walker L (1975) Procedural Justice: A Psychological Analysis. Erlbaum, Hillsdale, NJ.

[rego12323-bib-0073] Tyler TR (1994) Governing amid Diversity: The Effect of Fair Decision‐Making Procedures on the Legitimacy of Government. Law & Society Review 28, 809–831.

[rego12323-bib-0074] Tyler TR (2006) Psychological Perspectives on Legitimacy and Legitimation. Annual Review of Psychology 57, 375–400. 10.1146/annurev.psych.57.102904.190038 16318600

[rego12323-bib-0075] Ulbig SG (2008) Voice Is Not Enough. Public Opinion Quarterly 72, 523–539.

[rego12323-bib-0076] Van Ballaert B (2017) The European Commission's Use of Consultation during Policy Formulation: The Effects of Policy Characteristics. European Union Politics 18, 406–423.

[rego12323-bib-0078] West WF (2004) Formal Procedures, Informal Processes, Accountability, and Responsiveness in Bureaucratic Policy Making: An Institutional Policy Analysis. Public Administration Review 64, 66–80.

[rego12323-bib-0079] West WF (2009) Inside the Black Box. The Development of Proposed Rules and the Limits of Procedural Controls. Administration & Society 41, 576–599.

[rego12323-bib-0080] West WF, Raso C (2013) Who Shapes the Rulemaking Agenda? Implications for Bureaucratic Responsiveness and Bureaucratic Control. Journal of Public Administration Research and Theory 23, 495–519.

[rego12323-bib-0081] Wonka A, Rittberger B (2010) Credibility, Complexity and Uncertainty: Explaining the Institutional Independence of 29 EU Agencies. West European Politics 33, 730–752.

[rego12323-bib-0082] Yackee SW (2006) Sweet‐Talking the Fourth Branch: The Influence of Interest Group Comments on Federal Agency Rulemaking. Journal of Public Administration Research and Theory 16, 103–124.

[rego12323-bib-0083] Yackee SW (2014) Participant Voice in the Bureaucratic Policymaking Process. Journal of Public Administration Research and Theory 25, 427–449.

[rego12323-bib-0084] Yackee JW, Yackee SW (2006) A Bias Towards Business? Assessing Interest Group Influence on the U.S. Bureaucracy. Journal of Politics 68, 128–139.

[rego12323-bib-0085] Yee AS (1996) The Causal Effects of Ideas on Policies. International Organization 50(1), 69–108.

[rego12323-bib-0086] Young KL, Pagliari S (2015) Capital United? Business Unity in Regulatory Politics and the Special Place of Finance. Regulation & Governance 11, 3–23.

