# Supplementary material for: Stakeholder consultations and the legitimacy of regulatory decision‐making: A survey experiment in Belgium
Source: Regul Gov. 2020 Jun 11;15(3):877–93. doi: 10.1111/rego.12323 (PMC8359407; doi:10.1111/rego.12323)
Supplement: Supplementary file 1 — Appendix [file REGO-15-877-s001.pdf]

Online Appendix

Vignettes (in the original language)

Condition I: no consultation with stakeholders
Voedselverpakkingen duurder, maar milieuvriendelijker vanaf 2020

Het Europees milieuagentschap heeft een voorstel voor nieuwe regulering uitgewerkt die het gebruik van biologisch afbreekbare verpakkingen voor alle voedingsmiddelen zou verplichten vanaf 2020. Zowel de voedsel- en verpakkingsindustrie, als consumentenorganisaties betreuren het gebrek aan overleg bij de totstandkoming van het voorstel.

De regulering maakt deel uit van een pakket maatregelen om de CO_2 productie te reduceren en klimaatopwarming tegen te gaan. Bij de productie van biologisch afbreekbare materialen komt 70% minder CO_2 vrij ten opzichte van plastic verpakkingen.

De nieuwe regulering zou voor voedsel- en verpakkingsbedrijven grote kosten met zich meebrengen, aangezien de productie van biologisch afbreekbare materialen duurder is dan de productie van plastic. “Wij betreuren dat het milieuagentschap ons niet heeft betrokken bij de totstandkoming van dit voorstel, terwijl de goedkeuring ervan grote gevolgen zal hebben voor onze bedrijven”, zegt de woordvoerder van de Europese Federatie voor Voedselproducenten.

Ook enkele consumentenorganisaties reageren op het gebrek aan overleg: “Deze maatregel zal tot aanzienlijke prijsstijgingen leiden voor de consument. Wij vinden het dan ook jammer dat het milieuagentschap ons niet heeft geconsulteerd tijdens de voorbereidingen van het voorstel”, zegt een vertegenwoordiger van de Europese Consumentenorganisatie.

Condition IIa: only business
Voedselverpakkingen duurder, maar milieuvriendelijker vanaf 2020

Het Europees milieuagentschap heeft een voorstel voor nieuwe regulering uitgewerkt die het gebruik van biologisch afbreekbare verpakkingen voor alle voedingsmiddelen zou verplichten vanaf 2020. Bij de totstandkoming van het voorstel werden vertegenwoordigers van de voedsel- en verpakkingsindustrie geconsulteerd, als leden van een bestaande adviesraad binnen het milieuagentschap. Consumentenorganisaties daarentegen betreuren dat zij niet bij dit overleg betrokken werden.

De regulering maakt deel uit van een pakket maatregelen om de CO_2 productie te reduceren en klimaatopwarming tegen te gaan. Bij de productie van biologisch afbreekbare materialen komt 70% minder CO_2 vrij ten opzichte van plastic verpakkingen.

De nieuwe regulering zou voor voedsel- en verpakkingsbedrijven grote kosten met zich meebrengen, aangezien de productie van biologisch afbreekbare materialen duurder is dan de productie van plastic. “Toch zijn wij tevreden dat het milieuagentschap ons via de adviesraad heeft betrokken bij de totstandkoming van dit voorstel, aangezien de goedkeuring ervan grote gevolgen zal hebben voor onze bedrijven”, zegt de woordvoerder van de Europese Federatie voor Voedselproducenten.

Consumentenorganisaties reageren op het gebrek aan overleg met andere stakeholders dan de industrie: “Deze maatregel zal tot aanzienlijke prijsstijgingen leiden voor de consument. Wij vinden het dan ook

jammer dat het milieuagentschap ons niet heeft geconsulteerd tijdens de voorbereidingen van het voorstel", zegt een vertegenwoordiger van de Europese Consumentenorganisatie.

Condition IIb: only consumer NGOs
Voedselverpakkingen duurder, maar milieuvriendelijker vanaf 2020

Het Europees milieuagentschap heeft een voorstel voor nieuwe regulering uitgewerkt die het gebruik van biologisch afbreekbare verpakkingen voor alle voedingsmiddelen zou verplichten vanaf 2020. Bij de totstandkoming van het voorstel werden vertegenwoordigers van consumentenorganisaties geconsulteerd, als leden van een bestaande adviesraad binnen het milieuagentschap. De voedsel- en verpakkingsindustrie daarentegen betreuren dat zij niet bij dit overleg betrokken werden.

De regulering maakt deel uit van een pakket maatregelen om de CO_2 productie te reduceren en klimaatopwarming tegen te gaan. Bij de productie van biologisch afbreekbare materialen komt 70% minder CO_2 vrij ten opzichte van plastic verpakkingen.

De nieuwe regulering zou voor voedsel- en verpakkingsbedrijven grote kosten met zich meebrengen, aangezien de productie van biologisch afbreekbare materialen duurder is dan de productie van plastic. "Wij betreuren dat het milieuagentschap ons niet heeft betrokken bij de totstandkoming van dit voorstel, terwijl de goedkeuring ervan grote gevolgen zal hebben voor onze bedrijven", zegt de woordvoerder van de Europese Federatie voor Voedselproducenten.

Consumentenorganisaties zijn tevreden dat ze als leden van een adviesraad binnen het milieuagentschap nauw betrokken werden: "Deze maatregel zal tot aanzienlijke prijsstijgingen leiden voor de consument. Wij zijn dan ook blij dat het milieuagentschap ons heeft geconsulteerd tijdens de voorbereidingen van het voorstel", zegt een vertegenwoordiger van de Europese Consumentenorganisatie.

Condition IIc: NGO and business
Voedselverpakkingen duurder, maar milieuvriendelijker vanaf 2020

Het Europees milieuagentschap heeft een voorstel voor nieuwe regulering uitgewerkt die het gebruik van biologisch afbreekbare verpakkingen voor alle voedingsmiddelen zou verplichten vanaf 2020. Bij de totstandkoming van het voorstel, werden vertegenwoordigers van consumentenorganisaties en van de voedsel- en verpakkingsindustrie geconsulteerd, als leden van een bestaande adviesraad binnen het milieuagentschap.

De regulering maakt deel uit van een pakket maatregelen om de CO_2 productie te reduceren en klimaatopwarming tegen te gaan. Bij de productie van biologisch afbreekbare materialen komt 70% minder CO_2 vrij ten opzichte van plastic verpakkingen.

De nieuwe regulering zou voor voedsel- en verpakkingsbedrijven grote kosten met zich meebrengen, aangezien de productie van biologisch afbreekbare materialen duurder is dan de productie van plastic. "Toch zijn wij tevreden dat het milieuagentschap ons via de adviesraad heeft betrokken bij de totstandkoming van dit voorstel, aangezien de goedkeuring ervan grote gevolgen zal hebben voor onze bedrijven", zegt de woordvoerder van de Europese Federatie voor Voedselproducenten.

Ook consumentenorganisaties zijn tevreden dat ze als leden van een adviesraad binnen het milieuagentschap nauw betrokken werden: "Deze maatregel zal tot aanzienlijke prijsstijgingen leiden voor de consument. Wij zijn dan ook blij dat het milieuagentschap ons heeft geconsulteerd tijdens de voorbereidingen van het voorstel", zegt een vertegenwoordiger van de Europese Consumentenorganisatie.

Condition III: public consultation

Voedselverpakkingen duurder, maar milieuvriendelijker vanaf 2020

Het Europees milieuagentschap heeft een voorstel voor nieuwe regulering uitgewerkt die het gebruik van biologisch afbreekbare verpakkingen voor alle voedingsmiddelen zou verplichten vanaf 2020. Tijdens de totstandkoming van het voorstel organiseerde het agentschap een publieke online consultatie, waarbij iedereen die dat wilde zijn of haar mening te kennen kon geven via de website van het agentschap.

De regulering maakt deel uit van een pakket maatregelen om de CO_2 productie te reduceren en klimaatopwarming tegen te gaan. Bij de productie van biologisch afbreekbare materialen komt 70% minder CO_2 vrij ten opzichte van plastic verpakkingen.

De nieuwe regulering zou voor voedsel- en verpakkingsbedrijven grote kosten met zich meebrengen, aangezien de productie van biologisch afbreekbare materialen duurder is dan de productie van plastic. Ook voor consumenten kan dit tot aanzienlijke prijsstijgingen leiden.

“Aangezien de maatregel verregaande gevolgen zal hebben voor zowel producenten als consumenten van voedingsmiddelen, hebben we ervoor gekozen om via een publieke online consultatie op onze website iedereen de mogelijkheid te geven om input aan te leveren tijdens de voorbereiding van het voorstel”, aldus één van de experts van het Europees milieuagentschap.

Debriefing (in original language)

Bedankt voor uw deelname! Het krantenbericht dat u bij het begin van de survey heeft gelezen is volledig fictief. Er zijn vijf verschillende versies van het krantenbericht, waarin de mate van overleg met stakeholders varieert. Door de respondenten willekeurig één van de vijf versies te presenteren, vooraleer de vragenlijst wordt ingevuld, willen we testen of de mate van overleg met stakeholders een effect heeft op het draagvlak voor een beslissing. Indien u hierover nog vragen heeft mag u ons altijd contacteren via email@email.be.

Table A1. Dependent, independent and control variables

	Mean	SD	Min.	Max.
Procedure acceptance citizens (rescaled)	0.42	0.23	0	1
Procedure acceptance companies (rescaled)	0.35	0.23	0	1
Decision acceptance citizens (rescaled)	0.71	0.25	0	1
Decision acceptance companies (rescaled)	0.61	0.27	0	1
Left-right citizens	4.80	2.46	0	10
Left-right companies	5.68	1.99	0	10
Attitude towards consultation citizens (control)	3.57	0.72	1	5
Attitude towards consultation companies (control)	3.56	0.68	1	5

Design robustness and manipulation checks

During the past decades, an substantial number of experiments have been conducted in the field of political science and public administration (for reviews see Anderson & Edwards 2015; Blom-Hansen *et al.* 2015; Bouwman & Grimmelikhuijsen 2015; Margetts 2011). Most of these studies rely on student samples or on self-selected panels of individuals. As our subjects were part of a self-selected citizen panel, the results may not be representative of the broader population, which is part of a broader problem with the experiments' lack of external validity (Druckman *et al.* 2011). Indeed, experimental results are not always directly comparable to results obtained from representative randomized population samples in which all observations have a known probability of being sampled (statistical generalizability). Nevertheless, our study design has two features – one concerning the sample and another concerning the experimental design – that may partially alleviate some of these concerns. First, it is difficult to establish whether our response rates (46% for the citizen panel and 4.4% for the company leaders) reflect a representative view of the entire set of affected companies and individual citizens in Belgium. Nonetheless, with respect to the company leaders, a highly diverse set of company leaders representing small independent businesses (including café and restaurant owners with zero or just a few employees), small and medium enterprises with a handful of (five to fifteen) employees, and large companies (some with more than 10,000 employees) took part in the experiment. The response rate of 46% for the citizen panel is difficult to qualify as 'high' or 'low' as it is not easy to compare our response rate with surveys relying on one shot random population samples or with similar experiments that are mostly based on a convenience sample (many experimental studies do not report response rates). Response rates in one shot random population samples might be different (and/or somewhat higher) compared to a citizens panel where panel-members can decide whether or not to take part in a particular study or experiment. Moreover, panels are characterized by some attrition as some panel members drop out, but did not formally unsubscribe from the panel. Our response rate of 46% is still reasonable; the average response rate of the Antwerp citizen panel is 40%. In addition to this, we still have a sample that is large and diverse enough for our analyses. Although opt-in panels cannot be considered representative population samples, the citizen panel we use is more diverse than the panels used in experiments conducted among students. The Antwerp citizen panel seeks to maximize the diversity of subjects by attracting respondents from all corners of society, walks of life, and professional backgrounds (see Walgrave *et al.* 2009, pp. 158-159; for similar approaches see Esaiasson *et al.* 2017; Grimmelikhuijsen & Meijer 2014; de Fine Licht 2014a). Compared to the population at large, the subjects are more likely to be male (61%); they are highly educated (33% have university educations), and they demonstrate more interest in politics than the average citizen ($\bar{x} = 8.88$ on a scale ranging from 1 'not interested at all' to 11 'highly interested'; population $\bar{x} = 6.21$).[1] Although the average panel member is somewhat more leftist ($\bar{x} = 5.87$ on a scale ranging from 0 'right wing' to 10 'left wing'; population $\bar{x} = 5.14$) than an average citizen in the overall population, a substantial number of panel members are center-right or right-wing-leaning (56% of the panel voted for center-right, right or radical right-wing parties).

Second, our primary goal is to shed light on the causal relation between stakeholder consultations, procedure and decision acceptance while controlling for confounding factors (Bouwman & Grimmelikhuijsen 2015; Calder *et al.* 1982; Druckman *et al.* 2011; Morton & Williams 2010, pp. 322-351). Our aim is not to draw firm conclusions that apply to a wider population of cases (see conclusion) but rather to test the causal impact of varying consultation arrangements. Two design features enable us to conduct a robust test: 1) the use of a valid and reliable measurement instrument and 2) random assignment of subjects to the experimental conditions.

To begin, we designed the experimental conditions in the form of fictitious news articles, and these were pre-tested in a pilot phase (see paper). Also relevant for enhancing realism is that the subjects were not invited to an artificial laboratory environment but instead completed the questionnaire at home or in their work settings. In addition, the questionnaire included two questions that were designed to reveal

[1] To compare our sample with the population, we rely on the Belgian Partirep Electoral Study 2014 (see goo.gl/W2iCGD).

whether the experimental conditions worked as intended. Did respondents understand the vignettes correctly, implying that responses provided us with valid measures of what we aimed to test? These two questions were framed as follows:

1. *Based on this news article, to what extent do you think that there was room for input from stakeholders during the development of the proposal? By stakeholders, we mean anyone who will, directly or indirectly, bear the consequences of the regulation, for instance companies or consumers (Control 1).*
2. *Based on this news article, to what extent do you think there was room for input from only a limited group of stakeholders or from anyone? By stakeholders, we mean anyone who will, directly or indirectly, bear the consequences of the regulation, for instance companies or consumers (Control 2).*

The second question, which is a follow-up to the first, was not presented to the respondents who received the version of the questionnaire in which there was no stakeholder involvement. Both questions were answered on a seven-point scale ranging from 1 (‘no room for input’ or ‘a limited group of stakeholders’) to 7 (‘a lot of room for input’ or ’anyone interested could participate’); the responses reflect a gradation of stakeholder involvement ranging from weak to strong involvement. ANOVA-tests indicated that the vignettes worked as intended for the citizens (Control 1: $F_{(4,2133)} = 111.72$, $p = .000$; Control 2: $F_{(3,1698)} = 9.80$, $p = .000$) and for the companies (Control 1: $F_{(4,223)} = 5.67$, $p = .000$; Control 2: $F_{(3,175)} = 2.20$, $p = .089$). An overview of the mean values of the responses to the control questions for each vignette, divided with respect to citizens versus companies (Table A2). Only the public consultation condition worked differently than intended. We expected public consultations to be the most open form because they provide anyone who wishes to participate with the opportunity to do so. However, this was not reflected in our controls; namely, committees that included both business representatives and consumers were evaluated as showing a stronger stakeholder involvement than public consultations, while for this condition we would expect the highest values in response to both control questions. Two explanations may account for this. First, stakeholder involvement via public consultations might be less familiar to citizens or company leaders and therefore more difficult to judge. Second, other vignettes explicitly mentioned two types of stakeholders, namely business interests and/or consumer groups. The public consultation vignette reported that ‘anyone’ had the opportunity to provide comments. This result suggests that individuals may need more detailed information on who participated if they are to judge the quality of a consultation, specifically its openness to stakeholders (see paper).

Finally, our main goal was to test how the five experimental conditions, i.e., different consultation arrangements, influence procedure and decision acceptance. Ensuring that the measurements of the experimental conditions are truly independent observations requires that the assignment of subjects to these five conditions does not result in confounding factors. Random assignment implies that each subject has the same probability of experiencing the stimulus, which reduces the chance of statistically significant relations between relevant control variables and the dependent variable and allows us to test the net impact of the experimental conditions. More specifically, the procedure acceptance index and the decision acceptance index might be higher for respondents with higher political trust, higher internal efficacy and higher external efficacy (e.g., Almond & Verba 1963). Additionally, the general attitude towards stakeholder consultations is expected to affect procedure and decision acceptance. Finally, we controlled for four demographic variables: gender, age, professional background and level of education. Below we an overview of how these eight control variables were measured and Table A3 provides ANOVA- and Chi^2-tests. These tests indicate that the randomization worked well; no significant differences were observed among the five groups except for the variable ‘general attitude towards stakeholder consultations’ in the citizen sample ($F_{(4,2133)} = 5.29$, $p = .0001$).

Table A2. Mean values of the treatment control questions for each vignette

		Citizens		Companies	
		Control 1	Control 2	Control 1	Control 2
Condition I	no consultation	2.30	/	2.39	/
Condition IIa	committee only business groups	3.03	3.19	2.86	2.91
Condition IIb	committee only citizen groups	3.28	3.33	2.60	2.55
Condition IIc	committee business and citizen groups	4.30	3.71	3.81	3.42
Condition III	public consultation	3.69	3.63	3.08	3.08

Table A3. ANOVA and Pearson Chi^2 test results of the control variables

	Companies	Citizens
Political trust	F(4,208) = 1.10; p = 0.359	F(4,2133) = 1.53, p = 0.190
Internal efficacy	F(4,207) = 0.73; p = 0.572	F(4,2133) = 0.77; p = 0.542
External efficacy	F(4,207) = 0.03; p = 0.999	F(4,2133) = 0.32; p = 0.866
Attitude towards consultation	F(4,207) = 0.36; p = 0.835	F(4,2133) = 5.29; p = 0.000
Gender	chi2(4) = 0.78; p = 0.941	chi2(4) = 1.077; p = 0.898
Age	F(4,207) = 0.25; p = 0.910	F(4,2132) = 0.68; p = 0.609
Professional situation	/	chi2(28) = 28.41; p = 0.443
Level of education	chi2(24) = 15.49; p = 0.906	chi2(24) = 22.63; p = 0.542

Note: professional situation was only asked in the citizen questionnaire

References

Almond GA, Verba S (1963) *The Civic Culture: Political Attitudes and Democracy in Five Nations.* Princeton, Princeton University Press.

Anderson DM, Edwards BC (2015) Unfulfilled Promise: Laboratory experiments in public management research. *Public Management Review* 17, 1518-1542.

Blom-Hansen, J, Morton R, Serritzlew S (2015). Experiments in Public Management Research. *International Public Management Journal* 18, 151-170.

Bouwman R, Grimmelikhuijsen SG (2015) Experimental public administration 1992-2014. *International Journal of Public Sector Management* 29, 110-131.

Calder BJ, Phillips LW, Tybout AM (1982) The Concept of External Validity. *Journal of Consumer Research* 9, 240–244.

de Fine Licht, J (2014a). Transparency Actually: How Transparency Affects Public Perceptions of Political Decision-making. *European Political Science Review* 6, 309–330.

Druckman JN, Green DP, Kuklinski JH, Lupia A (2011) *Cambridge Handbook of Experimental Political Science.* Cambridge, Cambridge University Press.

Esaiasson P, Gilljam M, Persson M (2017) Responsiveness Beyond Policy Satisfaction: Does It Matter to Citizens? *Comparative Political Studies* 50, 739-765.

Grimmelikhuijsen SG, Meijer AJ (2012) Effects of Transparency on the Perceived Trustworthiness of a Government Organization: Evidence from an Online Experiment. *Journal of Public Administration Research and Theory* 24, 137–157.

Margetts HZ (2011) Experiments for Public Management Research. *Public Management Review* 13, 189-208.

Morton RB, Williams KC (2010) *Experimental Political Science and the Study of Causality. From Nature to the Lab.* Cambridge, Cambridge University Press.

Walgrave S, Lefevere J, Nuytemans M (2009) Issue Ownership Stability and Change: How Political Parties Claim and Maintain Issues Through Media Appearances. *Political Communication* 26, 163-172.

Operationalization of the control variables

a) *Political trust*

To what extent do you trust the following actors:

- Companies
- Consumer organizations
- Trade unions
- The legal system
- Political parties
- The federal parliament
- The federal government
- The European Union
- The European Central Bank

5-point Likert scale: Very untrustworthy, untrustworthy, neither trustworthy nor untrustworthy, trustworthy, very trustworthy

b) *Attitude towards stakeholder consultation*

Policymakers sometimes consult different stakeholders during the development of new policies. By stakeholders, we mean anyone who will be, directly or indirectly, affected by these policies, for instance companies or consumers. To what extent do you (dis)agree with the following statements:

- This slows down policymaking without added value.
- This ensures that all kinds of information and knowledge can better reach policymakers.
- This makes that those who are already in charge will gain even more influence.
- This ensures that difficult decisions will gain more societal support.

5-point Likert scale: Strongly disagree, disagree, neither agree nor disagree, agree, strongly agree

c) *Political efficacy*

To what extent do you (dis)agree with the following statements:

- Politicians are only interested in my vote and not in my opinion.
- People like me do not have any influence on politics.
- I consider myself well-qualified to participate in politics.
- I feel that I could do as good a job in public office as most other people.

5-point Liker scale: Strongly disagree, disagree, neither agree nor disagree, agree, strongly agree

d) *Socio-demographics*

- What is your gender?
  - Male
  - Female

- What is your date of birth?

- How would you characterize your professional situation?
  - Worker
  - Clerk
  - Manager
  - Independent/professional
  - Entrepreneur
  - Civil servant

- Student
- Other:

- What is your highest level of education?
  - Primary education
  - Lower secondary education
  - Higher secondary education
  - Professional Bachelor
  - Academic Bachelor or Master
  - Doctoral degree
  - Other:

Table A4. The effect of consultation arrangement on procedure and decision acceptance, with control variables (Hypothesis 1, 2, 3a, and 3b)

	procedure		decision	
	citizen<br>Model AI	company<br>Model AII	citizen<br>Model AIII	company<br>Model AIV
Intercept	0.31 (0.02)***	0.33 (0.06)***	0.70 (0.02)***	0.70 (0.06)***
Left-right	-0.06 (0.02)**	-0.13 (0.08)*	-0.23 (0.02)***	-0.19 (0.08)**
General attitude towards consultations	0.10 (0.03)***		0.20 (0.03)***	
Stakeholder consultation:				
I.no consultation (=ref.)	-	-	-	-
IIa.closed only business	0.01 (0.02)	0.09 (0.05)	-0.01 (0.02)	0.00 (0.06)
IIb.closed only consumers	0.07 (0.01)***	0.04 (0.05)	-0.00 (0.02)	-0.03 (0.06)
IIc.closed business and consumers	0.16 (0.01)***	0.18 (0.05)***	0.00 (0.02)	-0.05 (0.06)
III.public consultation	0.09 (0.02)***	0.13 (0.05)***	-0.02 (0.02)	0.06 (0.06)
R^2	0.08	0.09	0.08	0.04
F	31.10	4.44	32.15	1.57
Df	6	5	6	5
N	2138	219	2138	213

Note: all variables are scores to range from 0 to 1; unstandardized regression coefficients; standard errors in parentheses; * p<0.05 **, p<0.01, *** p<0.001

Table A5. Testing contrasts between slopes in Figure 1 (Hypothesis 4)

A. Average predicted decision acceptance by procedure acceptance and political orientation [95% Confidence Intervals]

	procedure acceptance (PA)		
	high≥0.6 (*n*=696)	medium >0.3 and <0.6 (*n*=711)	low≤0.3 (*n*=731)
political orientation			
left-right≤3 (most leftist, *n*=785)	0.88	0.76	0.67
	[0.87,0.88]	[0.76,0.77]	[0.66,0.67]
left-right=4, 5, and 6 (centrist, *n*=702)	0.84	0.70	0.58
	[0.83,0.84]	[0.70,0.71]	[0.57,0.58]
left-right≥7 (most rightist, *n*=651)	0.82	0.64	0.48
	[0.81,0.83]	[0.64,0.65]	[0.47,0.49]

B. Contrasting medium levels of PA with high and low levels (F, p, df=1)

	medium versus high PA	medium versus low PA
political orientation		
left-right≤3 (most leftist)	37.34, p<.0001	38.07, p<.0001
left-right=4, 5, and 6 (centrist)	41.16, p<.0001	56.20, p<.0001
left-right≥7 (most rightist)	50.43, p<.0001	54.89, p<.0001

Further robustness checks

Analysis of variance (ANOVA) and OLS-regression requires several assumptions. One particular assumption concerns heteroscedasticity or the constant distribution of the residuals. We have implemented various tests in order to check for heteroscedasticity (Levene for model I-IV reported in Table 3 and White for model V-VIII in Table 4). This Appendix also presents some additional models (for instance, Welch ANOVA and regression models with robust standard errors). As one can see these additional analyses did not change the results reported in the manuscript. With respect to the ANOVA-models in Table 3, our tests show that there is considerable heteroscedasticity in Model 1, much less in Model III, but not in Model II and Model IV. Concerning models V-VIII, we face potential heteroscedasticity problems in all four models. Levene- and White-tests are reported in Table A6.

Table A6. Overview Levene- and White-tests Models Reported in Table 3 an Table 4 (df=4)

Levene-test Table 3	F	p	Df
Model I	3.79	.0045	4
Model II	1.12	.3471	4
Model III	2.24	.0622	4
Model IV	1.30	.2722	4
White-test Table 4	Chi^2	p	Df
Model V	173.26	<.0001	5
Model VI	178.55	<.0001	8
Model VII	16.14	.0065	5
Model VIII	16.80	.0323	8

One of the reasons for this heteroscedasticity is that the variances, but especially the shape of the distribution differ substantially across the five treatments. For instance, in case of Treatment IIc individuals were very likely to show high levels of procedure and decision acceptance, which resulted in a negatively skewed distribution. Given the different nature of the treatments, it is not unlikely that in addition to the mean, also the variance will differ across treatments, which makes that heteroscedasticity is a typical problem for experimental studies. Although a problem with heteroscedastic data is that it may affect standard errors and related significance tests, quite a number of statisticians argue that ANOVA is pretty robust to deviations from the homogeneity assumption, especially if the data are balanced, which is the case in our design (see for instance Bathke 2004; Zimmerman 2004).

Table A7. Welch ANOVA-tests Models Reported in Table 3 (df=4)

	F	P
Model I	39.09	<.0001
Model II	5.56	.0004
Model III	0.71	.5837
Model IV	0.60	.6606

A feature of heteroscedasticity tests is that they are all-or-nothing tests, but that they do not tell us what to do if there is a potential problem with heteroscedasticity. Moreover, it is not because such tests indicate non-constant variance that statistical tests will always be biased or invalid. There are different ways to deal with such situations. We could transform the data, for instance implement a Box Cox transformation or normalize the data. We tested such models, with largely the same results, but one of the problems with these models is that transformations complicate the interpretation of the coefficients. Instead, we ran various robustness checks for the models reported in Table 3 and Table 4, namely we re-analyzed the data with Huber-White standard errors and (for the ANOVA in Table 3) we conducted a Welch's ANOVA-test. An analysis with Huber-White standard errors may lead to higher standard errors compared to what the normal

standard errors used to be, but if the two sets of standard errors do not differ significantly, we can be confident that the results are not biased. The Welch test is considered to be a more reliable test for ANOVA's where different treatments show unequal variance. These robustness checks (Table A7, A8 and A9) show almost identical results compared to the results we reported in the paper.

Table A8. Models I-IV with Huber-White standard errors

	procedure		decision	
	citizen<br>Model I	company<br>Model II	citizen<br>Model III	company<br>Model IV
Intercept	0.35 (0.01)***	0.25 (0.03)***	0.71 (0.01)***	0.60 (0.04)***
Stakeholder consultation:				
I.no consultation (=ref.)	-	-	-	-
IIa.closed only business	0.01 (0.01)	0.09 (0.05)	-0.09 (0.02)	-0.00 (0.06)
IIb.closed only consumers	0.08 (0.01)***	0.05 (0.05)	-0.00 (0.02)	-0.02 (0.06)
IIc.closed business and consumers	0.17 (0.02)***	0.19 (0.04)***	0.00 (0.02)	0.01 (0.06)
III.public consultation	0.09 (0.02)***	0.14 (0.04)**	-0.02 (0.02)	0.06 (0.06)
R^2	0.07	0.08	0.00	0.01
F	39.10	4.79	0.74	0.56
Df	4	4	4	4
N	2138	219	2138	219

Note: all variables are scores to range from 0 to 1; unstandardized regression coefficients; standard errors in parentheses; * p<0.05 **, p<0.01, *** p<0.001

Table A9. Models V-VIII with Huber-White standard errors

	individual citizens		company leaders	
	non-interactive<br>Model V	Interactive<br>Model VI	non-interactive<br>Model VII	Interactive<br>Model VIII
Intercept	0.60 (0.01)***	0.68 (0.02)***	0.55 (0.06)***	0.54 (0.09)***
Procedure acceptance (PA)	0.51 (0.02)***	0.33 (0.04)***	0.41 (0.07)***	0.45 (0.17)*
Left-right	-0.22 (0.02)***	-0.36 (0.04)***	-0.14 (0.08)	-0.12 (0.14)
Procedure PA×left-right		0.36 (0.08)***		-0.07 (0.29)
R^2	0.29	0.29	0.15	0.15
F	429.66	297.26	18.65	12.39
Df	2	3	2	3
N	2138	2138	213	213

Note: all variables are scores to range from 0 to 1; unstandardized regression coefficients; standard errors in parentheses; * p<0.05 **, p<0.01, *** p<0.001

Bathke A (2004) The ANOVA F test can still be used in some balanced designs with unequal variances and nonnormal data. *Journal of Statistical Planning and Inference* **126**, 416-422.

Zimmerman DW (2004) A note on preliminary tests of equality of variances. *British Journal of Mathematical and Statistical Psychology* **57**, 173-181.
